# A Multi‐Scale Computational Framework for Evaluating Wireless Neuromodulation Feasibility via Magnetoelectric Nanoparticles

**DOI:** 10.1002/advs.77667

**Published:** 2026-09-10

**Authors:** Marta Bonato, Elric Zhang, Emma Chiaramello, Serena Fiocchi, Giulia Suarato, Valentin Gantenbein, Nathalia Cancino‐Fuentes, Alejandro Suarez‐Perez, Maria V. Sanchez‐Vives, Salvador Pané, Marta Parazzini

**Affiliations:** ^1^ Istituto di Elettronica e di Ingegneria dell'Informazione e delle Telecomunicazioni (IEIIT) CNR Milano Italy; ^2^ Multi‐Scale Robotics Lab Institute of Robotics and Intelligent Systems ETH Zürich Zürich Switzerland; ^3^ Institut d'Investigacions Biomèdiques August Pi i Sunyer (IDIBAPS) Barcelona Spain; ^4^ Facultat de Medicina i Ciències de la Salut Universitat De Barcelona (UB) Barcelona Spain; ^5^ ICREA Barcelona Spain

**Keywords:** brain neuromodulation, computational framework model, electric fields, magnetoelectric nanoparticles

## Abstract

Magnetoelectric nanoparticles (MENPs) represent a promising approach for wireless, minimally invasive neuromodulation with submillimetre precision. This study presents a multi‐scale computational framework that bridges the gap from single‐nanoparticle magnetoelectric properties to tissue‐level electric field distributions, establishing a digital twin approach adaptable to different experimental platforms. The framework operates across three interconnected levels. (1). At the nanoscale, a 2D axisymmetric COMSOL model characterizes individual MENP response, integrating experimental data from cobalt ferrite cores (15–20 nm diameter) with barium titanate shells. Parametric analysis reveals that larger cores generate higher electric potentials due to increased magnetostrictive volume. (2). At the in vitro level, electromagnetic simulations replicate the experimental setup as a digital twin, establishing baseline field distributions and confirming that coil‐induced electric fields alone remain below neuromodulation thresholds. (3). At the tissue scale, a dipole‐based approach models collective MENP behavior at physiologically relevant concentrations (0.1% and 0.2% w/v). Results demonstrate that MENPs generate widespread electric fields exceeding 10 V/m (77%–92% tissue coverage) with localized hotspots surpassing 100 V/m, intensities sufficient for neural modulation and activation. The framework provides quantitative predictions for optimizing nanoparticle design and stimulation parameters, supporting the rational development of MENP‐based neuromodulation strategies.

## Graphical abstract

A multiscale computational framework links single‐nanoparticle magnetoelectric behavior to tissue‐level electric field distributions, integrating experimental data from nanoparticle characterization and in vitro stimulation setups. Applied to a cortical slice configuration, the framework predicts optimal MENP concentrations and stimulation parameters, guiding the development of MENP‐based neuromodulation strategies adaptable to different experimental platforms.

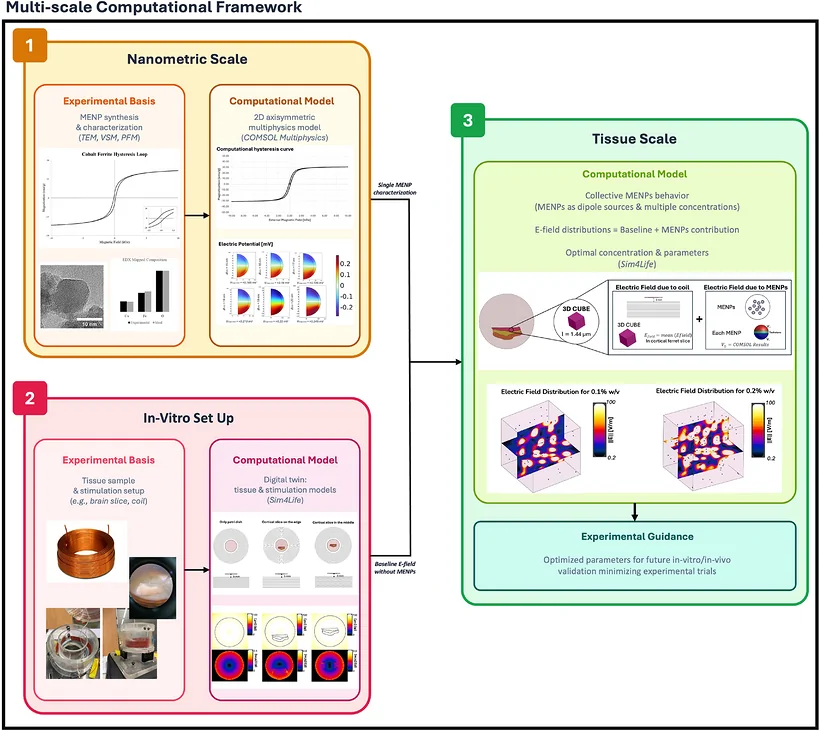

## 1 Introduction

The central nervous system (CNS) operates through complex patterns of electrical activity that can be disrupted by neurological and psychiatric disorders, ranging from stroke and chronic pain to Parkinson's disease and epilepsy. These disorders significantly impact quality of life, representing major health and economic problems [1]. Recording, understanding, and manipulating brain neural electrical signals has therefore become crucial for both diagnosis and treatment, becoming a central focus of numerous research initiatives worldwide [2].

Building on evidence that electric fields influence neuronal excitability, several stimulation techniques have been developed, many of which have shown clinical efficacy in treating neurological and psychiatric conditions by restoring normal electrical patterns in affected brain regions. Briefly, brain stimulation techniques can be classified as invasive and non‐invasive [3, 4]. Among traditional invasive approaches, implantable electrodes for deep brain stimulation (DBS) have been used for nearly three decades, primarily to treat certain forms of Parkinson's disease, and have also been studied for other applications. These electrodes provide excellent control over neural stimulation with millisecond temporal precision and sub‐millimeter spatial resolution. However, the invasiveness of electrode implantation presents significant risks, including infection, haemorrhages, potential need for hardware replacement, and long‐term complications from inflammatory responses [5].

To address these limitations, non‐invasive brain stimulation (NIBS) techniques have emerged in recent decades, eliminating surgical risks while still effectively modulating neural activity [6]. Among the NIBS techniques, methods such as Transcranial Magnetic Stimulation (TMS) and transcranial current stimulation (tDCS and tACS) show promise across multiple therapeutic applications from pain management to stroke recovery [7, 8]. Nevertheless, these techniques also face their own constraints: wireless stimulation is weakened as it passes through skin and cranial tissues, restricting effects to shallow cortical areas, typically not exceeding 2 cm in depth [9]. Furthermore, their spatial precision remains considerably limited when compared to electrode‐based technologies, with stimulation fields extending across multiple centimetres [4, 10].

This has motivated the search for hybrid approaches that overcome the constraints of both invasive and non‐invasive techniques, combining the precise spatial resolution of electrode‐based methods with the safety and accessibility of wireless stimulation.

Recent advances in magnetoelectric (ME) nanostructures offer a promising solution toward achieving this integration, potentially enabling wireless, minimally‐invasive neuromodulation with submillimetre precision (<1 mm) while avoiding the disadvantages associated with traditional methods [11, 12, 13, 14]. In ME nanostructures, two distinct material phases, one magnetostrictive and one piezoelectric, are coupled at their interface, enabling the conversion of magnetic field into electric field through a mechanical deformation: when an external magnetic field is applied, the magnetostrictive component undergoes dimensional changes (strain) due to the reorientation of its magnetic domains. This mechanical deformation is transferred through the interface to the piezoelectric phase, which responds to mechanical stress by generating an electric polarization and, consequently, a localized electric field. Through this magneto‐mechano‐electric coupling, magnetic stimulation can remotely induce localized electric fields at the nanoscale, forming the basis for potential neural modulation [11, 15].

The most widely used ME materials employ a core–shell geometry (Magnetoelectric Nanoparticles, i.e. MENPs) with a magnetostrictive core typically CoFe_2_O_4_ (CFO) or Fe_3_O_4_ (FO) encapsulated by a piezoelectric BaTiO_3_ (BTO) shell. This architecture offers several strategic advantages for brain modulation applications: nanoscale dimensions (15–50 nm) facilitate blood‐brain barrier crossing, the BTO shell ensures biocompatibility minimizing inflammatory responses and tissue damage, and the system generates highly localized electric fields in response to low‐frequency (1–1000 Hz) and low‐intensity (10–150 mT) magnetic flux density (B), enabling safe, prolonged protocols [16, 17, 18].

Research studies have demonstrated MENPs effectiveness across in vitro, ex vivo, and in vivo models, showing direct neuronal modulation with rapid response times (<1s), enhanced spatial resolution, and wireless synchronization of neural activity [19, 20]. In vivo studies have confirmed blood‐brain barrier crossing [21], remote behavioral control when targeted to relevant brain regions, and minimal inflammatory response [20].

In parallel, to better understand, support, and guide the experimental findings, computational studies have emerged investigating both the magnetoelectric behavior of individual MENPs and the effects of MENPs clusters distributed in neural tissue. Regarding the literature on single MENP studies, computational frameworks have been developed to characterize magnetoelectric coupling coefficients, optimize particle geometry and composition, and predict electric field generation [22, 23, 24, 25]. These models provide valuable insights into how parameters such as core–shell dimensions, particle shape, and magnetic field characteristics affect the performance of individual nanoparticles.

As a complementary approach, other modelling studies focused on collective MENPs behavior, by using cluster of MENPs, simulating the distributions and effects of nanoparticles within neural tissue [26, 27, 28]. More recently, other computational studies focused on the quantification of the influence of the concentration and distribution of MENPs in peripheral nerve for regeneration or activation purpose [29, 30].

**FIGURE 1:**
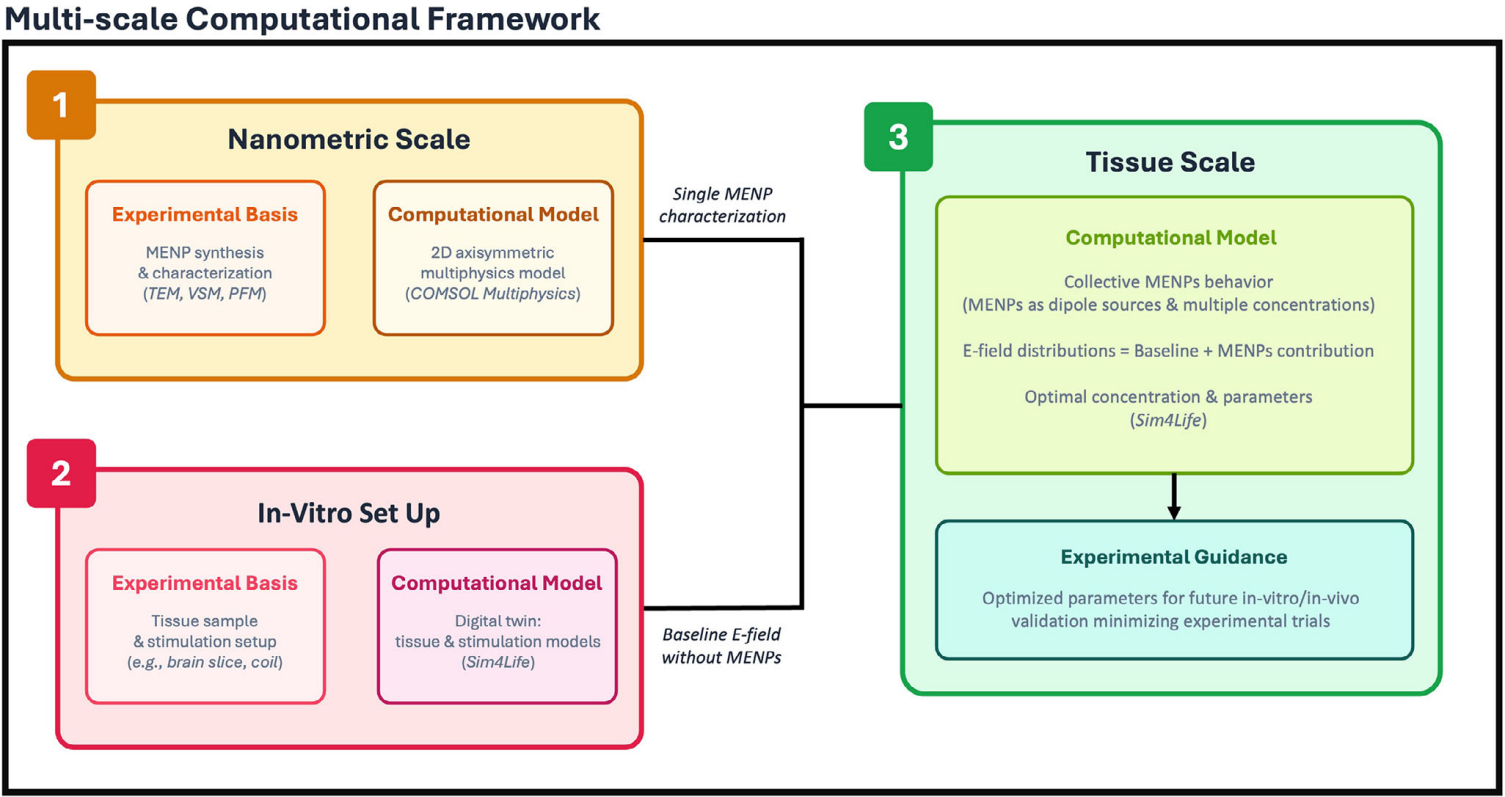
Multi‐scale computational framework for MENPs‐based neuromodulation. The framework integrates three interconnected levels. At the nanometric scale (1) and in vitro setup level (2), experimental characterization data provide the basis for developing and validating computational models of single‐MENP response and baseline tissue electric fields, respectively. At the tissue scale (3), a computational model simulates collective MENP behavior to predict electric field distributions at different concentrations. This predictive capability optimizes parameters for future in vitro and in vivo validation, minimizing experimental trials.

Building on these computational works, this study develops a comprehensive multi‐scale modelling framework (Figure 1) to assess whether MENPs can generate electric fields of sufficient magnitude to influence neuronal activity under safe magnetic stimulation conditions, bridging the gap from single‐nanoparticle magnetoelectric properties to tissue‐level electric field distributions. The framework establishes a digital twin approach that can be adapted to different experimental platforms; here, we demonstrate its application to an in vitro configuration consisting of cortical slices exposed to coil‐generated magnetic fields, but the methodology applies to any MENP‐based system once the single‐particle shell polarization is characterized.

The computational framework operates across three interconnected levels, where the first two levels integrate experimental data with computational modelling, while the third level provides purely predictive capabilities to guide future experiments. At the nanometric scale (Level 1), building on our previous modelling study [23], we developed a 2D axisymmetric model in COMSOL Multiphysics to characterize individual MENP magnetoelectric response. This model implements fully coupled multiphysics simulations integrating magnetic field interactions, magnetostrictive deformation, and piezoelectric field generation. Simulations were based on experimental characterization data from core–shell nanoparticles with spherical CFO cores (15–20 nm diameter), including geometric dimensions and hysteresis magnetization curves. The model allows the quantification of core magnetization values and electric potential levels on the shell surface.

At the in vitro setup level (Level 2), we developed an electromagnetic model replicating the experimental tissue sample and stimulation setup to characterize baseline magnetic and electric field distributions within cortical tissue in the absence of nanoparticles, enabling validation against measurable quantities before proceeding to predictive simulations.

At the tissue scale (Level 3), to evaluate the effect of MENPs introduction, we implemented a dipole‐based approach to represent collective MENP behavior, adapting the framework from [30]. Each MENP is modelled as an electric dipole, with potential values derived from single‐particle simulations at Level 1. This strategy enables simulation of realistic nanoparticle distributions at physiologically relevant concentrations (0.1% and 0.2% w/v, corresponding to approximately 100–200 nanoparticles per simulation volume), including random spatial positioning to capture realistic dispersion patterns, while avoiding the computational costs of full multiphysics modelling for hundreds of individual particles. The model quantifies volumetric electric field distributions throughout the cortical tissue, enabling statistical analysis of stimulation coverage and identification of regions exceeding neural modulation and activation thresholds (1–100 V/m) [31].

This integrated approach generates experimental guidance by predicting optimal MENP concentrations and stimulation parameters, helping identify optimal MENP parameters and clarify the mechanisms driving neural modulation. While demonstrated here for a specific in vitro configuration, the framework provides a generalizable platform where different experimental setups can be modelled, supporting the rational design of MENP‐based neuromodulation strategies.

## 2 Results

### 2.1 Fabrication and Characterization of Core–Shell MENPs

Magnetoelectric core–shell nanoparticles (MENPs) were synthesized using previously established methods [19, 32], to create a consistent baseline for neural experiments. For a typical MENP, cobalt ferrite (CoFe_2_O_4_) cores of 15–20 nm in size were created following a standard wet‐chemical coprecipitation route based on nitrate salts. The cobalt ferrite cores were then coated with barium titanate (BaTiO_3_) following a sol‐gel to autocombustion process with citric acid as the chelating agent and mediator to ensure homogeneous shell formation and controlled thickness.

**FIGURE 2:**
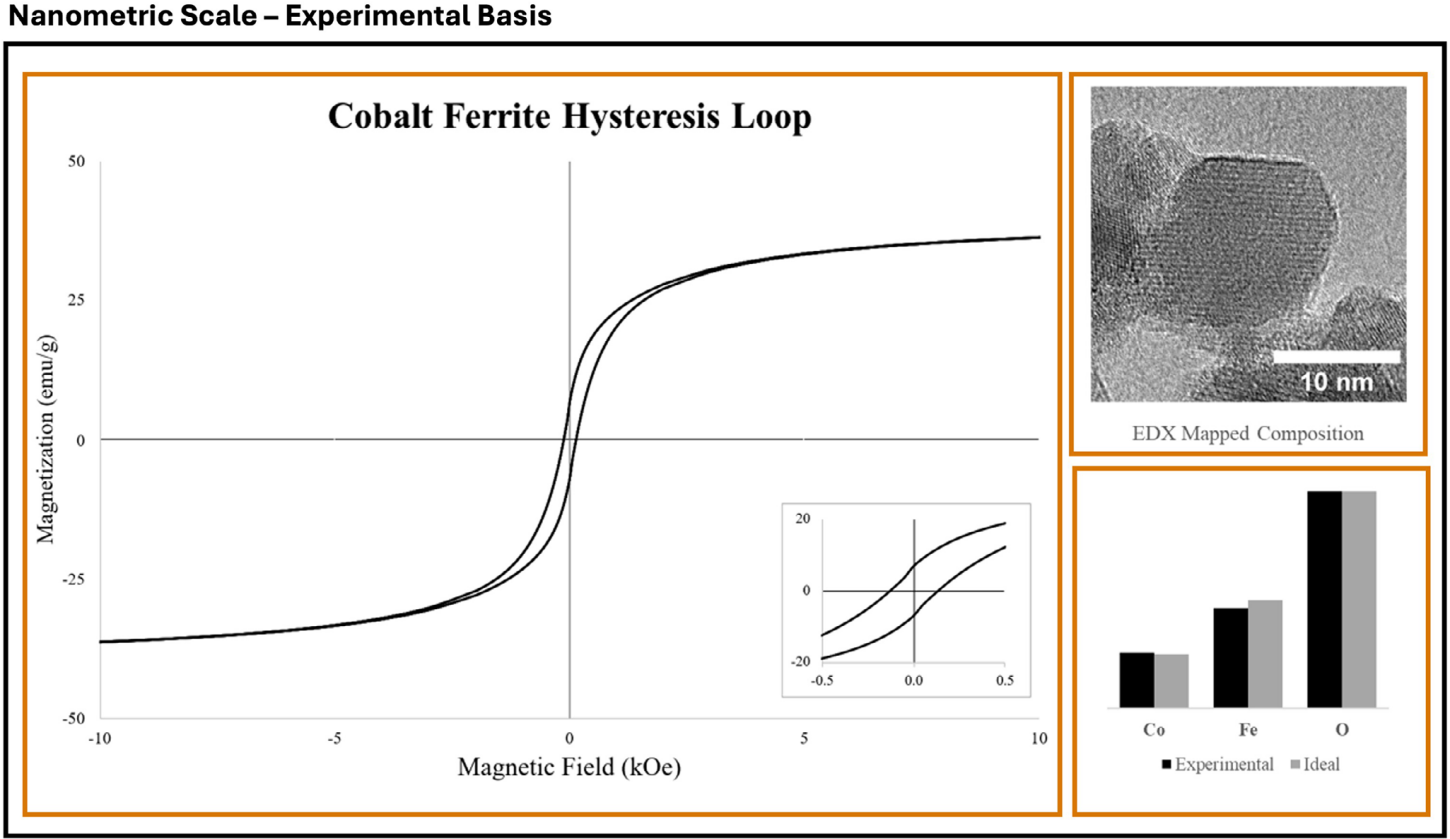
Experimental Characterization: magnetization curve of the cobalt ferrite nanoparticles and low field regime (inset), HR‐TEM image of a cobalt ferrite core with clear crystallinity, and EDX‐derived composition data.

The resulting core–shell nanoparticles were rigorously characterized to determine physical parameters critical for modeling their magnetoelectric response. Experimentally derived values (illustrated in Figure 2) included the nanoparticle's physical dimensions, captured through transmission electron microscopy (TEM), magnetic behavior, measured through vibrating sample magnetometry (VSM), and the estimated magnetoelectric coefficient, tested via piezoforce microscopy (PFM). Additional TEM images showing multiple nanoparticles and the crystalline structure of a single core are reported in Figure S1. These experimentally determined parameters were then integrated into the multiphysics model to estimate electrical behavior in neuromodulatory simulations.

### 2.2 Computational Model Validation and Single MENP Magnetoelectric Characterization

**FIGURE 3:**
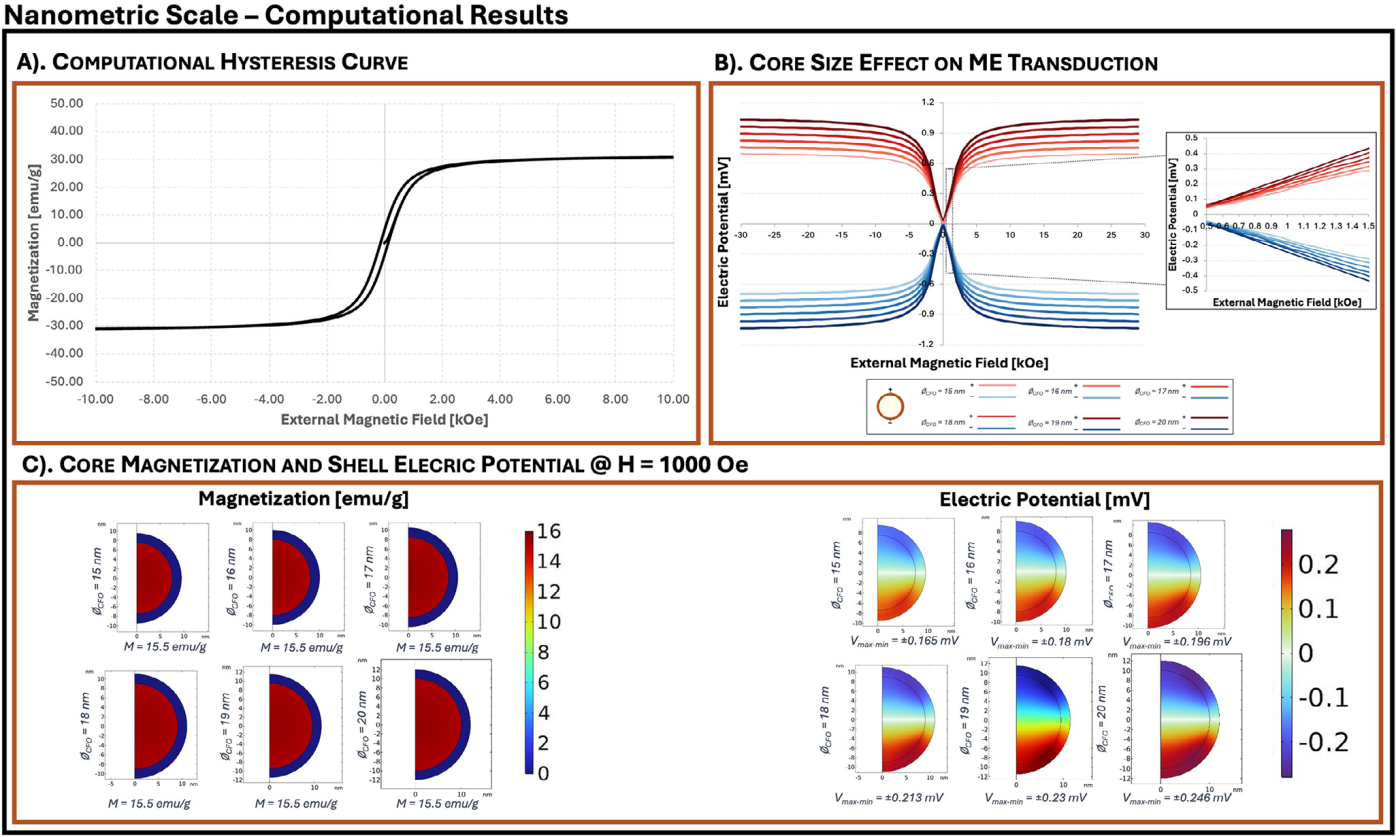
Computational results from 2D axisymmetric COMSOL model for spherical core MENPs. (A) Hysteresis characterization: magnetization curve of the magnetostrictive CFO core. (B) Core size effect on magnetoelectric transduction: electric potential generated on the BTO shell surface as a function of external magnetic field for core diameters ranging from 15 to 20 nm. (C) Representative 2D distributions showing core magnetization (left) and shell electric potential (right) at H = 1000 Oe operating field strength for the investigated core sizes.

Figure 3A presents the hysteresis curve obtained from the 2D computational model for the spherical core geometry. The curve was generated by varying the external magnetic field in the range of −10–+10 kOe and computing the corresponding core magnetization at each field intensity. The CFO core magnetization exhibited characteristic hysteresis behavior, ranging from approximately −30 to +30 emu/g. When compared with the experimental hysteresis curve shown in Figure 2 the computational results demonstrate excellent agreement, accurately reproducing both the coercive field and saturation magnetization values. This strong correspondence validates our computational model for spherical core MENPs.

To account for the variability in nanoparticle fabrication, we systematically varied the spherical CFO core diameter from 15 to 20 nm in 1 nm increments while maintaining a constant 2 nm BTO shell thickness. Figure 3B shows the electric potential at the two opposite poles of the BTO shell as a function of external magnetic field up to saturation, for each core diameter. Larger cores generate systematically higher potential differences across the entire field range, with saturation values increasing from ±0.70 mV (Φ_CFO_ = 15 nm) to ±1.04 mV (Φ_CFO_ = 20 nm). Figure 3C presents the spatial distributions at H = 1000 Oe, corresponding to a B field of 100 mT in air, the value selected for tissue‐level simulations. All configurations exhibit the same core magnetization (15.5 emu/g), while the shell surface potential increases from ±0.165 mV (Φ_CFO_ = 15 nm) to ±0.246 mV (Φ_CFO_ = 20 nm). This size‐dependent enhancement results from the increased magnetostrictive volume, which generates greater total mechanical deformation transferred to the piezoelectric shell. To realistically represent the size variability typical of nanoparticle synthesis, tissue‐level simulations use the average electric potential across the investigated core sizes (i.e., ±0.205 mV at B = 100 mT).

### 2.3 Baseline Electromagnetic Field Characterization of the In Vitro Set Up

**FIGURE 4:**
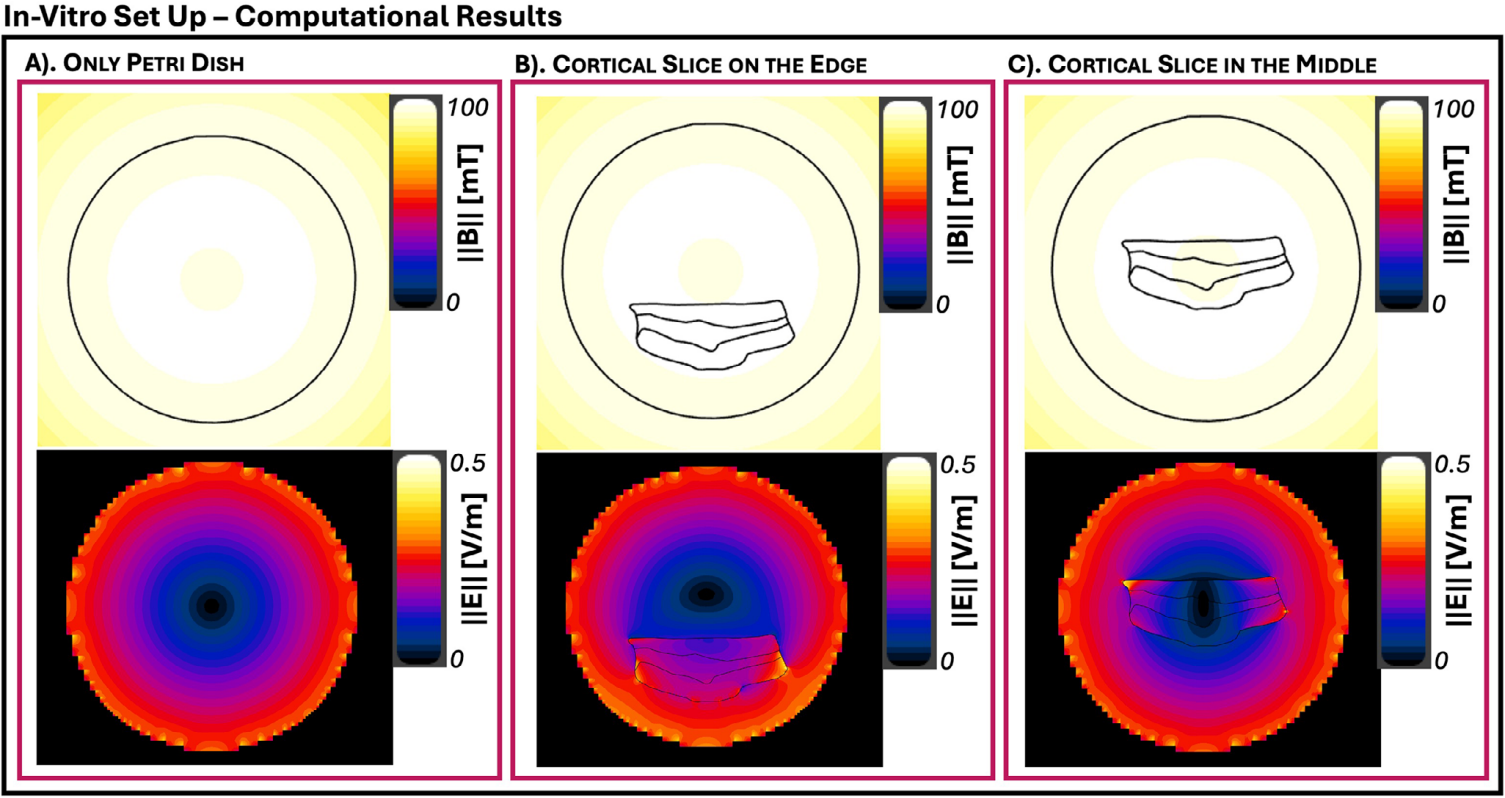
Magnetic flux density ||B|| [mT] (top row) and induced electric field ||E|| [V/m] (bottom row) distributions in the representative in vitro configuration. (A) Petri dish only. (B) Cortical slice positioned at the edge of the dish. (C) Cortical slice positioned in the middle of the dish.

To establish baseline electromagnetic conditions for the multiscale framework, we modelled a representative in vitro configuration consisting of a cortical slice positioned within a Petri dish exposed to coil‐generated magnetic fields, as shown in Figure 4. While specific to this geometry, the analysis illustrates how baseline field distributions can be characterized for any experimental setup before evaluating MENPs contributions.

Three simulation scenarios were compared: only the petri dish filled with extracellular fluid (Figure 4A), a cortical slice positioned at the edge of the petri dish (Figure 4B), and a cortical slice positioned in the middle of the petri dish (Figure 4C). Since the cortical slice may shift from its intended position during the experiment, two extreme placements were considered: the centre of the petri dish, representing the ideal configuration, and the edge, representing the worst‐case scenario in terms of field levels. This comparative analysis was designed to assess the spatial homogeneity of the B field and to quantify the variability of the induced electric field distribution as a function of slice position.

As expected, the B field distribution remained unchanged between the three configurations (Figure 4A–C, top row), maintaining excellent spatial homogeneity within the region of interest with constant values of approximately 100 mT throughout the petri dish volume in all scenarios.

In contrast, the induced electric field exhibited distribution and amplitude changes between the configuration without (only petri dish) and the two configurations with the cortical brain slice (Figure 4A–C, bottom row). In the configuration with only the petri dish containing extracellular fluid, the electric field displayed as a radially symmetric distribution with maximum values of 0.41 V/m localized at the outer edges of the petri dish. The introduction of the cortical slice changes this concentric pattern, particularly at the tissue boundaries. When the slice is positioned at the edge, dielectric distortions with maximum values of 0.47 V/m are observed, representing an approximately 15% increase compared to the only petri dish scenario. When the slice is positioned in the middle of the chamber, a localized peak electric field value of 0.55 V/m is observed in a very small region at the tissue boundaries, representing an approximately 33% increase compared to the first scenario. These edge effects are attributed to the conductivity mismatch between the extracellular fluid (σ = 2.68 S/m) and the brain tissue (σ = 0.35–0.42 S/m), which creates local field concentrations at the tissue‐fluid interface. However, the average electric field throughout the tissue volume was significantly higher when the cortical slice was in the edge position (0.20 V/m) compared to the central position (0.13 V/m). Moreover, also the central field values are lower when the slice was positioned in the middle, and the electric field distribution is less disturbed, showing improved spatial homogeneity. These baseline characterizations serve as the reference for evaluating the collective MENPs effect in Section 2.4, allowing the coil‐induced electric field to be distinguished from the localized enhancement produced by the nanoparticles. For the subsequent multi‐scale analysis, the higher average value of 0.20 V/m was conservatively adopted as the baseline contribution.

### 2.4 Cumulative MENPs Effects Through Multiscale Computational Framework

**FIGURE 5:**
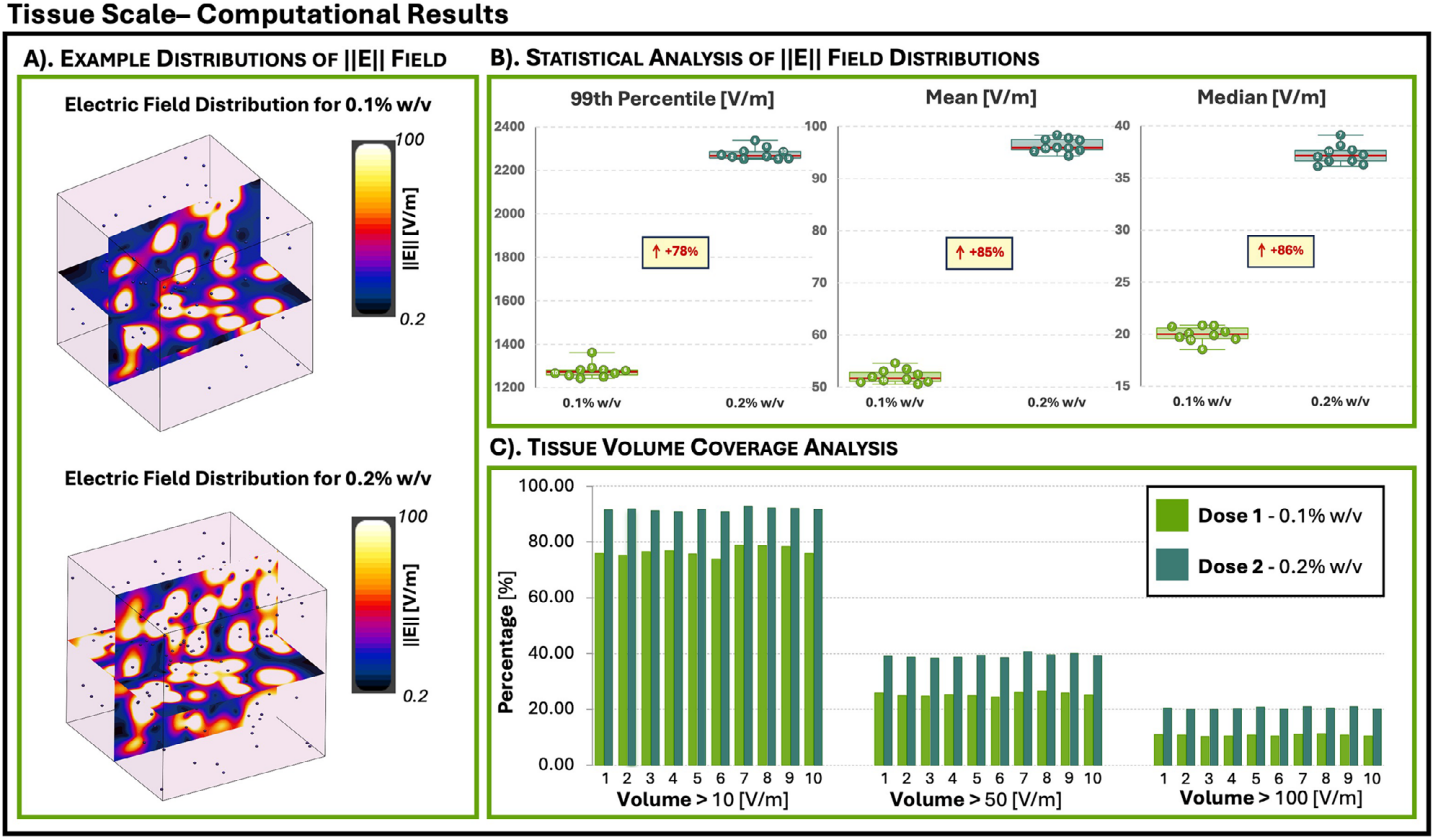
Multiscale computational framework results for collective MENP behavior in cortical gray matter. (A) Example spatial distribution of electric field generated by collective MENP behavior: Top: 0.1% w/v concentration (100 MENPs). Bottom: 0.2% w/v concentration (200 MENPs). The dots indicate the spatial positions of individual MENPs within the tissue. (B) Statistical analysis of electric field distributions across the two concentration scenarios (light green: 0.1% w/v, dark green: 0.2% w/v). The boxplots represent the variability across ten random configurations, while the numbered markers indicate the electric field values for each individual configuration. (C) Tissue volume coverage analysis for clinically relevant neural modulation thresholds (light green: 0.1% w/v, dark green: 0.2% w/v). The bar charts show the percentage of tissue volume with electric field strength above three critical thresholds: >10, >50, and >100 V/m. Numbers 1–10 represent the ten different analyzed MENP spatial configurations.

An example of electric field distributions generated by collective MENPs interacting within brain gray matter is visualized in Figure 5A, taken from the first configuration of the ten random distributions. It is important to note that these total electric field distributions experienced by the tissue are the sum of the coil baseline contribution alone (0.20 V/m, as described in Section 2.3) plus the local MENP‐induced enhancement shown in the figure. On the left, at 0.1% w/v concentration (100 MENPs), localized electric field hotspots emerged around individual nanoparticles, with peak intensities near the particle surfaces. Furthermore, the field intensity rapidly decayed with distance, in the range of nanometre scale, exhibiting the characteristic dipolar spatial profile. On the right, at 0.2% w/v concentration (200 MENPs), the increased particle density resulted in a more complex field distribution characterized by better spatial coverage and increased overlap between adjacent particle fields, leading to larger regions experiencing higher electric field amplitude. This increase can be explained by the higher probability of MENPs being closer together at higher concentrations. Indeed, although the electric field generated by each MENP decays sharply within nanometres of its surface, field magnitude at any point is governed primarily by proximity to the nearest particle(s). In this way, as concentration increases, so does the chance of neighbouring MENPs fields overlapping, producing an increase in the upper range of the electric field distribution. This is further consisted with the quantitative analysis in Figure 5B,C.

Figure 5B shows the statistical analysis for electric field values across all ten random distributions for both concentration scenarios. As it can be seen from the figure, the 99th percentile electric field increased from approximately 1280 V/m at 0.1% w/v to approximately 2280 V/m at 0.2% w/v, representing a +78% increase. The mean electric field demonstrated an +85% increase, rising from approximately 52 to 96 V/m. The median electric field exhibited the most pronounced relative change with an +86% increase, from approximately 20 to 37 V/m. Interestingly, the box plot distributions revealed consistent statistical spread across the ten realizations, with variations coefficient values below 3% in all the metrics, indicating robust behavior, despite the randomly MENPs placement.

The volume coverage analysis, presented in Figure 5C, quantifies the tissue fraction experiencing electric fields above clinically relevant neural modulation thresholds. For the >10 V/m threshold, the tissue coverage was approximately 77% and 92% for 0.1% and 0.2% w/v, respectively, a small increase, consistent with coverage already approaching since coverage at this threshold was already extensive at both concentrations. On the other hand, substantial concentration‐dependent effects were also observed at higher field thresholds: the >50 V/m coverage increased from 25% to 39%, and the >100 V/m coverage increased from 11% to 20% for 0.1% and 0.2% w/v, respectively, reflecting that where coverage starts lower, the increase with concentration is almost double. These results indicate that doubling the MENP concentration not only increases the mean field strength but also expands the tissue volume experiencing high‐magnitude electric fields capable of inducing neural activation.

## 3 Discussion

This study introduces a multi‐scale modeling framework to investigate the feasibility of wireless neuromodulation based on magnetoelectric nanoparticles (MENPs), addressing whether these nanoparticles can generate electric fields of sufficient magnitude to influence neuronal activity and how the scale gap from single‐particle properties to neural tissue‐level effects can be bridged. By creating a digital twin approach adaptable to different experimental platforms, grounded in experimental data, we have successfully modelled the system across multiple scales: from single MENP behavior at the nanoscale, through the in vitro cerebral cortex slices, up to the collective effects of MENPs in generating electric fields within biological brain tissue. This computational framework provides quantitative insights for designing minimally invasive neuromodulation strategies and can guide future experimental investigations.

Compared to existing computational approaches for MENP‐mediated stimulation, this framework offers several methodological advances. Previous studies have typically addressed either single‐particle magnetoelectric behavior [22, 23, 24, 25] or tissue‐level field distributions [26, 27, 28] as separate problems, whereas our approach integrates both scales within a unified workflow where single‐particle outputs directly inform tissue‐level simulations. Furthermore, while earlier collective models often relied on idealized particle arrangements or simplified dipole clusters, we explicitly account for random spatial distributions and evaluate variability across multiple configurations, providing statistically robust predictions. Finally, the framework incorporates experimentally characterized nanoparticles, rather than relying solely on theoretical material parameters. Together, these features make the framework adaptable to different experimental platforms and capable of guiding nanoparticle optimization for specific applications.

The first stage of the computational framework, the single‐MENP model, relied on experimental characterization data. To ensure reproducibility and scalability, wet‐chemical synthesis methods were prioritized for producing large batches of nanoparticles with uniform properties, establishing a consistent baseline for neurological experiments. Nanoparticle characterization combined high‐resolution single‐particle techniques (TEM, PFM) with ensemble measurements (XRD, VSM), providing a comprehensive understanding of both individual and collective properties. Once the target dimensions and material characteristics were achieved, key fabrication parameters (i.e., core and shell dimensions from TEM imaging, magnetic properties from VSM measurements) were integrated into the 2D axisymmetric model. It is worth noting that these magnetic parameters were derived from measurements on the synthesized nanoparticles themselves, and therefore intrinsically account for any size‐ or surface‐related contribution to their magnetic response. In this way, the model accurately reproduced the experimentally measured magnetic hysteresis curve for MENPs with a spherical core, confirming the validity of the 2D model implementation and allowing to confidently explore the impact of core geometry on magnetoelectric performance. The parametric analysis of spherical core diameter (15–20 nm) revealed that core size significantly influences magnetoelectric performance. Increasing core diameter from 15 to 20 nm enhanced the electric potential generated on the BTO shell surface by approximately 49% at the operating H field of 1000 Oe. This size‐dependent enhancement stems from the increased magnetostrictive volume, which generates greater total mechanical deformation transferred to the piezoelectric shell. This finding demonstrates that core size represents a critical design parameter for maximizing stimulation efficiency while maintaining dimensions compatible with blood‐brain barrier crossing (all configurations < 25 nm total diameter).

The second stage of the framework models the in vitro system configuration. As described in Section 4.2, this represents one specific implementation of MENP‐mediated stimulation; however, the methodology is designed to be adaptable to any MENP‐based experimental setup. Once tissue geometry, coil specifications, and dielectric properties are defined, the same computational approach can characterize electromagnetic field distributions for alternative configurations, including different coil designs, tissue preparations, or stimulation chambers. This adaptability constitutes a core strength of the framework, enabling optimization of diverse in vitro systems on a case‐by‐case basis. A fundamental requirement for MENP‐mediated stimulation is a homogeneous B field across the tissue volume, ensuring uniform nanoparticle activation. The simulations confirmed this condition: B field homogeneity within the tissue remained high, with a coefficient of variation below 1%, as expected given the negligible magnetic permeability contrast between brain tissue and surrounding media. In contrast, the induced electric field distribution showed strong dependence on tissue dielectric properties, with field enhancement occurring at boundaries. Notably, while extremely low‐frequency B fields in the µT to mT range have been reported to influence CNS function, although with subtle and poorly reproducible effects [33], even with an applied B field of 100 mT, the induced electric field remains below 1 V/m, at least one order of magnitude lower from the 1 to 10 V/m threshold required for reliable neuromodulation [31]. This finding confirms that, in the absence of MENPs, the low‐frequency coil‐induced electric field alone is incapable of inducing significant neuromodulation, highlighting the indispensable role of the nanoparticles for wireless neural modulation.

Finally, in the third and most predictive step, the analysis of the collective behavior of MENPs based on an electric dipole approach combined with the coil baseline contribution in Sim4Life software revealed the ability of these nanoparticles to generate electric fields at neuromodulation‐relevant intensities [34, 35, 36]. At experimentally relevant concentrations (0.1% and 0.2% w/v), simulations revealed widespread fields exceeding 10 V/m capable of modulating neuronal firing, alongside localized hotspots exceeding 100 V/m, intensities comparable to TMS and DBS intensities capable of direct neuronal activation [37]. MENP concentrations could therefore be used as a design parameter, expanding the suprathreshold volume at higher concentrations while the already widespread subthreshold coverage grows comparatively little, offering a way to control the balance between diffuse subthreshold modulation and focal suprathreshold activation. Furthermore, since the simulation domain functions as a representative elementary volume, these field patterns can be spatially replicated to characterize the electric field environment experienced by larger neuronal structures. However, how widespread subthreshold modulation and localized suprathreshold activation interact at the network level remains to be investigated.

Placing our results in perspective with current neurostimulation techniques, the MENP‐based approach offers potential advantages over existing methods. Unlike DBS, which requires invasive neurosurgery with associated risks, MENPs can potentially be delivered through minimally invasive routes to reach deep brain structures. Compared to TMS, which is limited to superficial cortical regions (2–3 cm depth) with spatial resolution of 1–2 cm due to rapid field decay, and to tDCS, which generates diffuse cortical fields with limited focality (several centimetres), MENPs could theoretically enable deep penetration with sub‐millimeter precision defined by nanoparticle distribution. These characteristics position MENP‐mediated stimulation as a potentially transformative technology bridging the gap between the deep reach but invasiveness of DBS and the safety but limited focality of non‐invasive techniques, though translation to clinical practice requires systematic validation studies [11, 12, 13, 14].

However, it is important to acknowledge the limitations inherent to this computational study. While our validated modelling framework provides valuable quantitative predictions, electrophysiological validation will be essential to fully characterize the neuromodulator effects of MENPs in physiological conditions. The model represents a first‐order approximation that does not yet account for potential nanoparticle agglomeration, their detailed interaction with cell membranes, or the full complexity and heterogeneity of the cellular microenvironment. Direct experimental confirmation of MENP spatial distribution within the tissue at high resolution was not performed in this study; related core–shell MENP formulations have shown measurable tissue penetration and retention via TEM and fluorescence imaging in organotypic slice preparations [38], and applying similar imaging approaches to the present system represents an important direction for future work. Future studies should also model the interaction between these field distributions and realistic neuronal morphologies to fully capture spatially‐dependent effects on membrane polarization. In conclusion, our integrated computational framework represents a powerful tool for accelerating the development of MENP‐based neuromodulation. By demonstrating the ability to optimize nanoparticle design and predict their efficacy in neural tissue, this work helps lay the foundation for a new generation of wireless, precise, and minimally invasive neurological therapies.

## 4 Experimental Section

### 4.1 Experimental Fabrication Procedure for MENPs

For a comprehensive description of the synthesis methodology, please refer to our companion article [39], which addresses the material preparation in detail. In brief, a typical synthesis involves the addition of stoichiometric quantities of hydrolyzed cobalt and iron nitrate, individually added to separate vials of DI water under constant magnetic stirring. Once completely dissolved, the vials are then mixed and brought to the reaction temperature of 90°C under constant magnetic stirring. The precipitating agent sodium hydroxide, is then added to bring the pH to 10 and initialize the formation of cobalt ferrite nanoparticles. After the solution evolves for 30 min, the resulting nanoparticles are then magnetically separated and washed with DI water and ethanol.

The cobalt ferrite cores are then prepared for shell addition, with some set aside for characterization. For shelling, the cores are dispersed into DI water and treated with citric acid to improve monodispersion. The shell components of barium carbonate and titanium isopropoxide are dissolved in DI water and ethanol, respectively, before chelating with citric acid. Once all components are completely dissolved, the vials are mixed and reduced under constant magnetic stirring until a gel forms. This gel is then dehydrated and sintered at 700°C to produce the resultant core–shell magnetoelectric nanoparticles. For characterization, specific elements were necessary to produce useful modeling. We determined that nanoparticle geometry and magnetic hysteresis behavior (saturation and initial susceptibility) were the most critical factors. As such this required TEM imaging to determine single nanoparticle scale core and shell geometry and VSM characterization to understand the magnetic behavior and low field and saturation regimes.

### 4.2 In Vitro Cortical Slice Preparation and In Vitro Set Up

Several in vitro configurations can be employed to investigate MENP‐mediated neuromodulation, as long as a homogeneous B field is delivered across the tissue. Here, we present one representative setup based on acute cortical slices.

All procedures were carried out in compliance with Spanish (RD53/2013) and European (2010/63/EU) animal welfare regulations. Coronal slices (approximately 400 µm thick) containing cortical gray and white matter were obtained from adult ferrets following standard preparation protocols. Slices were maintained in an interface‐style chamber at 35°C–36°C and continuously perfused with carbogenated artificial cerebrospinal fluid (95% O_2_/5% CO_2_) to ensure oxygenation and pH stability throughout the experiment.

A custom stimulation coil was positioned beneath the chamber to deliver a uniform B field of 100 mT across the slice. The coil‐tissue distance was minimized while preserving proper humidity and perfusion conditions required for stable slice viability. MENPs were deposited onto the cortical gray matter surface using a controlled deposition protocol at two concentrations: 0.1% and 0.2% w/v (two droplets of 0.1% w/v equivalent to 6 µg per dose), ensured localized particle distribution and reduced lateral spread across the tissue.

This experimental configuration, with its realistic tissue geometry and experimentally relevant parameters, provides a reference scenario for the computational model for in vitro systems described in Section 5.2.

## 5 Computational Methods

### 5.1 Computational Model of a Single MENP

**FIGURE 6:**
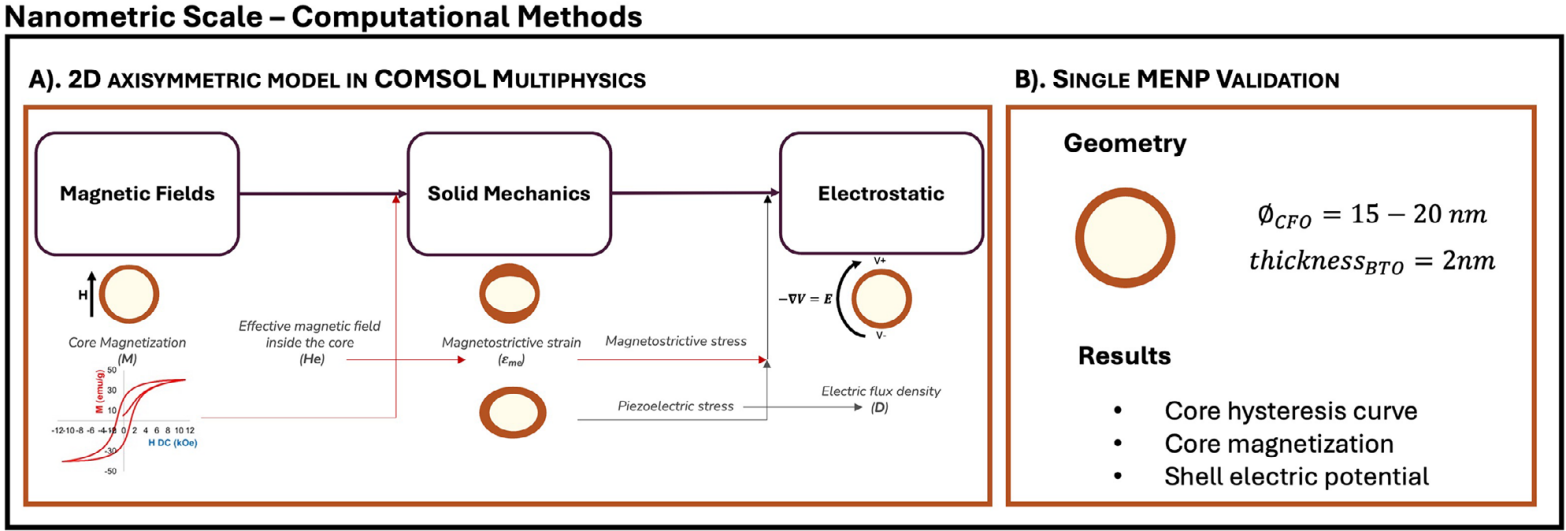
Single MENP Computational Model: (A). Two‐dimensional axisymmetric computational model of a single MENP implemented in COMSOL Multiphysics, integrating three coupled physics modules: Magnetic Fields, Solid Mechanics, and Electrostatics. (B). Validation results: core hysteresis behavior and shell electric potential distribution for the examined spherical core geometries (Ø = 15–20 nm).

To characterize the magnetoelectric response of individual MENPs and validate the computational approach against experimental measurements, following the methodology adopted in [22, 23], an axisymmetric 2D computational model was built using COMSOL Multiphysics software version 6.2 (https://www.comsol.it). The complete mathematical framework is illustrated in Figure 6A, where the 2D Multiphysics simulation integrated three physics modules: the Magnetic Fields module, the Solid Mechanics module, and the Electrostatics module. In the Magnetic Fields module, thanks to the use of the Jiles‐Atherton model [40], the core magnetization was computed. By adding the Solid Mechanics module, the magnetic field induces magnetostrictive strain, evaluating both magnetostrictive strain and resultant stress. The mechanical deformation triggers the piezoelectric effect in the BTO shell, modelled in the Electrostatics module, computing the resulting electric flux density and potential distribution on the MENP shell. Further details of the used parameters and governing equations of the 2D model are reported in the Supporting Information [23, 41]. As shown in Figure 6B, the computational model was applied to spherical CFO cores with diameters ranging from 15 to 20 nm (with 1 nm increments), each one surrounded by a 2 nm BTO shell. As previously described, these small dimensions ensure the crossing of the blood‐brain barrier (BBB), which typically allows passage of particles smaller than 50 nm, while the BTO shell was selected for its good biocompatibility in biological environments [20]. The parametric analysis of core size was designed to account for the dimensional variability observed during nanoparticle fabrication (Section 2.1), while investigating how such variations affect ME coupling efficiency. The model enabled the evaluation of core hysteresis curve by varying the externally applied magnetic field, as well as quantification of the core magnetization and the electric potential generated on the shell surface when subjected to an external B field strength within the range of low‐power magnetic stimulation used for in vitro brain modulation studies [31]. In this last case, the model was solved as a stationary study at H = 1000 Oe, corresponding to the B field equal to 100 mT generated in air by the coil when driven at 100 Hz, as show in Section 5.2. The electric potential values define the dipole source amplitudes used in Section 5.3.

### 5.2 Computational Model of the In‐Vitro System

**FIGURE 7:**
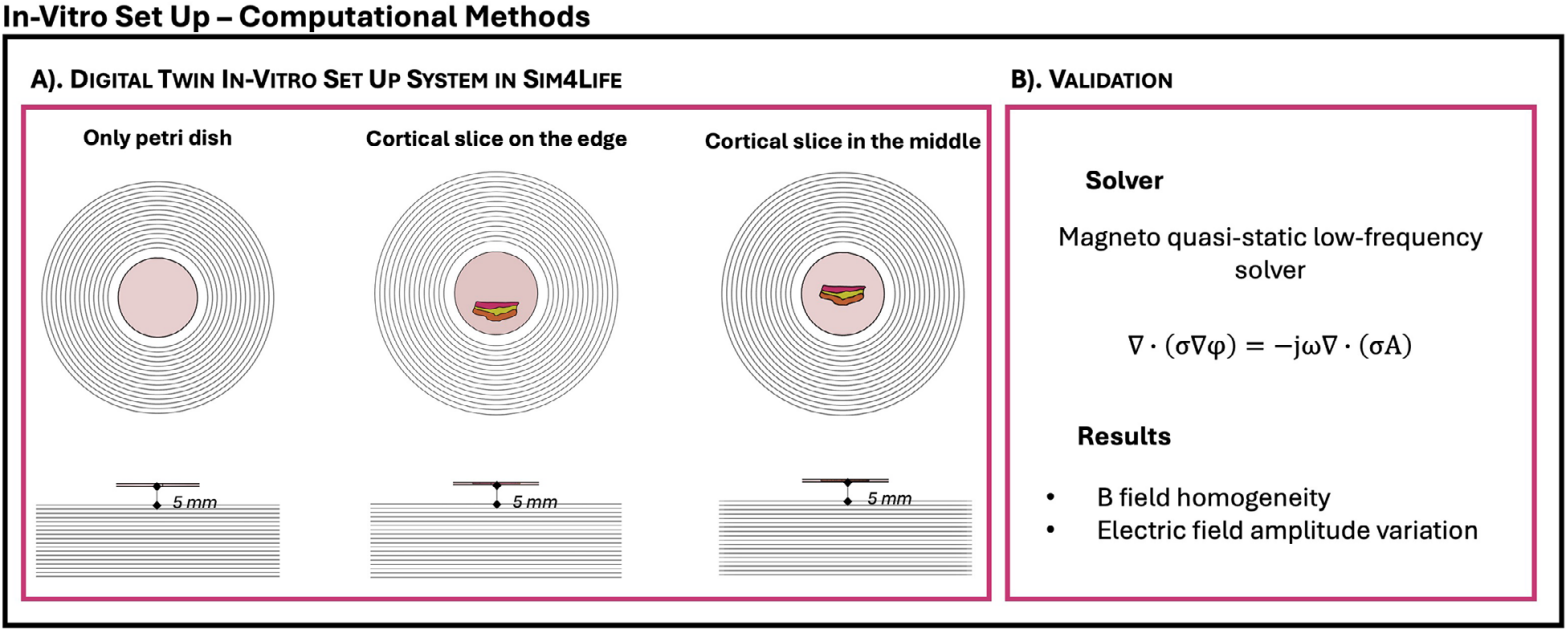
Computational model of the representative in vitro configuration. (A) Three simulation scenarios: petri dish only (left), cortical slice positioned at the edge of the dish (center), and cortical slice positioned in the middle of the dish (right). The top row shows the top view of the coil‐dish system; the bottom row shows the lateral view with the 5 mm coil‐dish distance. (B) Validation results: B‐field homogeneity across the region of interest and baseline electric field amplitude in the absence of nanoparticles.

To establish baseline electromagnetic field distributions before evaluating MENPs contributions, we developed a computational model of the representative in vitro configuration of Section 4.2, as illustrated in Figure 7A. The model consists of a stimulation coil with an inner diameter larger than the petri dish (2 cm diameter), containing extracellular fluid placed at 5 mm distance from the coil, and a cortical slice (placed inside the petri dish. The slice thickness (400 µm) and other geometric parameters are consistent with standard in vitro preparations and reflect possible realistic experimental values, as described in Section 4.2. However, the framework can be readily adapted to different configurations by updating the corresponding model inputs for any in vitro set up. The dielectric properties of the surrounding fluid were assigned as those of extracellular fluid (σ = 2.68 S/m and ε_r_ = 4.42 × 10^6^ at 100 Hz), while the cortical slice was modeled with distinct regions of gray and white matter, each with their respective dielectric properties at the same frequency (brain gray matter: σ = 0.42 S/m and ε_r_ = 3.91 × 10^6^ and brain white matter: σ = 0.35 S/m and ε_r_ = 1.67 × 10^6^ at 100 Hz) [42].

Simulations were conducted using the magneto‐quasi‐static low‐frequency solver in the simulation platform Sim4Life (https://sim4life.swiss), thanks to the Biot–Savart solver combined with the scalar potential finite‐element (SPFE) method. The operating frequency was set at 100 Hz, matching the excitation frequency used in the experimental coil setup. In this low‐frequency regime, where the computational domain dimensions are much smaller than the free‐space wavelength, the magnetic vector potential A decouples from the electric field E. Furthermore, since conduction currents dominate over displacement currents in biological tissues under these conditions, E can be derived from the scalar potential φ, which is given by:

$$\nabla \cdot \;\left(\sigma \nabla \varphi \right)=-j\omega \nabla \cdot \left(\sigma A\right)$$

where σ (Sm^−1^) represents the tissue conductivity and ω is the angular frequency of the field. The vector potential A is computed using the Biot–Savart's law, while φ is obtained through the finite‐element analysis. Three simulation scenarios were performed: only the petri dish filled with extracellular fluid, the cortical brain slice positioned at the edge of the petri dish, and the cortical slice positioned in the middle of the petri dish. This comparative analysis was designed to assess the spatial homogeneity of the B field with a peak amplitude of 100 mT within the region of interest and to quantify the spatial variability of the induced electric field distribution as a function of the tissue position in the petri dish (Figure 7B). These simulations establish the baseline electric field distribution in the cortical slice in the absence of nanoparticles, which serves as the reference for evaluating the collective MENPs effect described in Section 5.3. The mean electric field value computed in the cortical tissue is indeed incorporated into the multiscale framework in Section 5.3 to account for the coil contribution to the total field distribution when MENPs are present.

### 5.3 Computational Model of MENPs Collective Behavior in Biological Tissue

**FIGURE 8:**
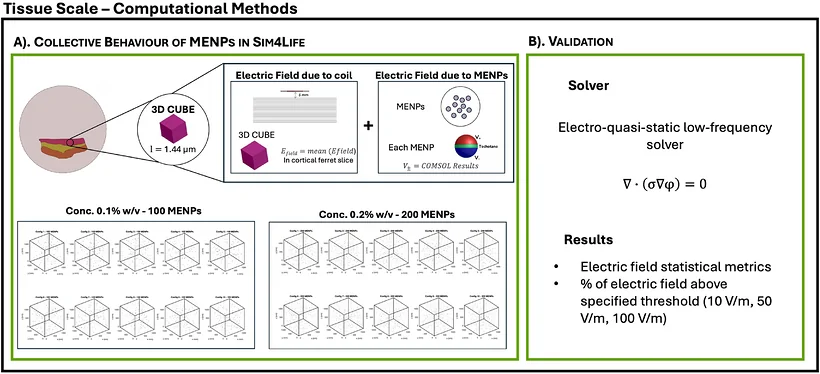
Multiscale computational framework for modeling collective MENP behavior in cortical gray matter. (A) Simulation approach: the cortical slice region of interest is discretized into cubic domains (1.44 µm side length), where the total electric field results from the superposition of the baseline coil‐induced field and the localized fields generated by MENPs modelled as electric dipoles. Ten independent random spatial configurations are shown for 0.1% w/v (100 MENPs, bottom left) and 0.2% w/v (200 MENPs, bottom right) concentrations. (B) Validation results: field distribution metrics and percentage of tissue volume exceeding clinically relevant thresholds (10, 50, and 100 V/m).

As shown in Figure 8A, the collective behavior of MENPs in cortical tissue was investigated by integrating results from the COMSOL single‐particle simulations with a multiscale computational framework adapted from previous studies [29, 30]. This approach allows the prediction of the electric field distributions generated by a multitude of MENPs under realistic experimental conditions, superimposed on the baseline coil‐induced electric field computed in Section 5.2.

Using the electromagnetics Sim4Life software (https://sim4life.swiss), each MENP was modelled as an electric dipole consisting of two oppositely charged hemispheres separated by an insulating Tecothane layer. The electric potential of the Tecothane layer was set to zero, while the electric potentials of the two hemispheres were assigned as the mean value derived from the parametric COMSOL simulations across the core size range (15–20 nm) described in Section 5.1. Two concentration scenarios were investigated: 0.1% and 0.2% w/v (Figure 8A, bottom row). These values were selected to match realistic experimental conditions (as described in Section 4.2), where nanoparticles are typically delivered onto the cortical surface via sequential droplet deposition, with 0.1% w/v representing a single dose and 0.2% w/v a cumulative two‐dose application. Particle positions were randomly assigned within the volume, reflecting the uncontrolled spatial arrangement of MENPs resulting from experimental droplet deposition onto the cortical slice; a minimum inter‐particle distance was imposed to reflect experimental conditions, in which nanoparticle agglomeration is actively avoided. In contrast, the orientation of each MENP dipole was assumed to be the same across all particles, aligned with the magnetic field generated by the coil, since the magnetostrictive core tends to align with the direction of an applied magnetic field. Random spatial positioning was therefore combined with a fixed, field‐aligned dipole orientation for all particles within each simulation volume. Furthermore, to capture the stochastic nature of particle dispersion and ensure statistical robustness, ten independent random distributions were generated for each concentration scenario. Each simulation domain consisted of a cubic volume of 1.44 µm side length selected to balance computational cost with adequate statistical sampling of MENPs number. The 3D volume has the dielectric properties of brain gray matter (σ = 0.42 S/m and ε_r_ = 3.91 × 10^6^) at 100 Hz. The 0.1% w/v concentration corresponded to 100 randomly positioned nanoparticles per simulation volume, while the 0.2% w/v concentration contained 200 nanoparticles. Although this volume is smaller than the one occupied by the pyramidal neurons in cortical slices, the simulation domain functions as a representative elementary volume. Given the homogeneous dielectric properties of cortical gray matter and the highly localized nature of MENP‐generated electric fields, which decay within nanometres from each particle surface, the computed field distributions can be spatially replicated to characterize the overall electric field environment experienced by larger neuronal structures.

Electric field distributions were computed using the low‐frequency (LF) electro‐quasistatic approximation implemented in Sim4Life software, evaluated at the same operating frequency of 100 Hz used in the coil model (Section 5.2). Under this approximation, ohmic currents dominate over displacement currents, and capacitive effects are neglected. The electric potential φ satisfies the Laplace equation, solved via the finite element method:

$$\nabla \cdot \left(\sigma \nabla \varphi \right)=0$$

where σ (Sm^−1^) represents the electrical conductivity of the tissue and φ denotes the electric potential. The electric field (E) distributions were obtained using the following relation:

E=−∇φ

The total electric field obtained represents the sum of the baseline coil‐induced field (computed as the mean value from the simulations in Section 5.2) and the localized electric fields generated by the presence of MENPs.

The resulting electric field distributions were characterized through quantitative metrics (Figure 8B), such as statistical measurements including the 99th, 75th, and 25th percentiles, median, and mean, providing insight into the field magnitude distribution across the tissue volume. Additionally, volume coverage analysis quantified the percentage of tissue experiencing electric fields exceeding clinically relevant thresholds of 10, 50, and 100 V/m. These threshold values were selected based on reported field strengths capable of inducing neural modulation or activation in the literature [31].

## Author Contributions

**Marta Bonato**: conceptualization, software, data curation, formal analysis, writing – original draft, writing – review and editing, methodology, investigation, validation. **Elric Zhang**: writing – review and editing, validation, data curation. **Emma Chiaramello**: conceptualization, writing – original draft. **Serena Fiocchi**: conceptualization, writing – original draft. **Giulia Suarato**: conceptualization, writing – original draft. **Valentin Gantenbein**: data curation, validation. **Nathalia Cancino‐fuentes**: writing – review and editing. **Alejandro Suarez‐perez**: writing – review and editing. **Maria V. Sanchez‐vives**: conceptualization, funding acquisition, writing – review and editing. **Salvador Pané**: conceptualization, writing – review and editing. **Marta Parazzini**: supervision, conceptualization, methodology, writing – original draft, writing – review and editing.

## Funding

This work was supported by the European Union's Horizon Europe programme through the European Innovation Council (EIC) r under grant agreement No 101130650 (META‐BRAIN).

## Conflicts of Interest

The authors declare no conflicts of interest.

## Supporting information




**Supporting File**: advs77667‐sup‐0001‐SuppMat.docx.

## Data Availability

The data that support the findings of this study are available from the corresponding author upon reasonable request.
