# Supplementary material for: A Multi‐Scale Computational Framework for Evaluating Wireless Neuromodulation Feasibility via Magnetoelectric Nanoparticles
Source: Adv Sci (Weinh). 2026 Sep 10:e77667. Online ahead of print. doi: 10.1002/advs.77667 (PMC13562792; doi:10.1002/advs.77667)
Supplement: Supplementary file 1 [file ADVS-9999-e77667-s001.docx]

**Supporting Information**

**Figure S1.** Transmission electron microscopy images of the synthesized MENPs. (A) Images of multiple nanoparticles, showing their spherical nature and approximate size. (B) Image of a single nanoparticle, showing its size and shape. Scale bars as indicated in each panel.

*Table I. MENPs Core properties*

|  | **Symbol** | **CFO** |
| --- | --- | --- |
| Core Diameter (nm) | *Ø_core_* | 15 - 20 |
| Magnetic saturation (A/m) | *M_s_* | 1.65*10^5^ |
| Magnetization reversibility | *c* | 0.001 |
| Domain wall density (A/m) | *a* | 2.3*10^4^ |
| Interdomain coupling | *α* | 0.001 |
| Pinning loss (A/m) | *k* | 1.1*10^4^ |
| Saturation magnetostriction (ppm) | *λs* | -200 |
| Density (kg/m^3^) | *ρ* | 5200 |
| Initial magnetic susceptibility | *Χ_0_* | 3 |
| Electrical conductivity (S/m) | *σ* | 4.2*10^-5^ |
| Poisson ratio | *ν* | 0.48 |
| Young’s modulus (GPa) | *E* | 230 |

*Table II. Shell properties*

|  | **Symbol** | **BTO** |
| --- | --- | --- |
| Shell Thickness (nm) | *t_shell_* | 2 |
| Density (kg/m^3^) | *ρ* | 5700 |
| Relative permittivity | *{ε_r11_; ε_r22_; ε_r33_}* | *{115.1;115.1_;_ 1251.3}* |
| Electrical conductivity (S/m) | *σ* | 1*10^-7^ S/m |
| Elasticity matrix, Voigt notation (GPa) | *{cE11; cE12; cE22; cE13; cE23; cE33; cE14; cE24; cE34; cE44; cE15; cE25; cE35; cE45; cE55; cE16; cE26; cE36; cE46; cE56; cE66}* | *{150.4;65.6;150.4;65.; 65.9; 145.5; 0; 0;0;43.9;0; 0; 0; 0;43.9; 0; 0; 0; 0; 0; 42.4}* |
| Piezoelectric coupling matrix, Voigt notation (C/m^2^) | *{eES11; eES21; eES31; eES12; eES22; eES32; eES12; eES23; eES33; eES14; eES24; eES34; eES15; eES25; eES35; eES16; eES26; eES36}* | *{0; 0; -4.32; 0; 0; -4.32;*  *0; 0; 17.4; 0; 11.4; 0;*  *11.4; 0; 0; 0; 0; 0}* |

**COMSOL Multiphysics models**

The single MENP model was implemented in COMSOL Multiphysics using three coupled physics modules: Magnetic Fields, Solid Mechanics, and Electrostatics. The governing equations for each module are described below.

**Magnetic Field Module**

The magnetic field physics was computed by solving Ampère's law:

$\nabla\times H=J$	(1)

$B=\nabla\times A$	(2)

$J=\sigma E$	(3)

together with the constitutive relationship:

$B=\mu_{0}(H+M)$	(4)

Where $H$ is the magnetic field, $B$ is the magnetic flux density, $A$ is the magnetic vector potential, $J$ is the current density, $E$is electric field and $M$ the magnetization.

The magnetization of the cobalt ferrite core was described using the Jiles-Atherton model, which decomposes the total magnetization into reversible ($M_{rev}$) and irreversible ($M_{irr}$) components:

$M=M_{rev}+M_{irr}$	(5)

is governed by a differential equation that accounts for domain wall pinning, anhysteretic magnetization, and the effective magnetic field.

**Solid Mechanics Module**

The mechanical response was obtained by solving the equation of motion and strain-displacement relationship:

$0=\nabla\cdot S+F_{v}$	(6)

$\varepsilon=\frac{1}{2}\left[ \left( \nabla u \right)^{T}+\nabla u \right]$	(7)

Where $S$ is the stress tensor, $F_{v}$ is the volume force, $u$ is the displacement, and $\varepsilon$ is the strain tensor.

For the magnetostrictive core, stress and strain were related by Hooke's law, incorporating magnetostrictive strain. Indeed, the magnetostrictive strain (ε_me_) of an isotropic material is expressed as:

$\varepsilon_{me}=\frac{3\lambda_{s}}{\mu_{0}M_{s}^{2}}dev\left( M\otimes M \right)$	(8)

where $\lambda_{s}$ is the saturation magnetostriction coefficient and $M_{s}$is the saturation magnetization

For the piezoelectric shell, the constitutive equation includes both elastic and piezoelectric contributions:

$S=S_{0}+C: \varepsilon+E\cdot e$	(9)

where $\boldsymbol{e}$ is the piezoelectric Voigt coupling matrix representing the stress tensor, $\boldsymbol{C}$ is the elastic right Cauchy deformation tensor and E is the electric field which is computed by using the piezoelectric coupling.

**Electrostatic Module**

The electric potential distribution was computed by solving Gauss's law:

$\nabla\cdot D=\rho_{v}$	(10)

$E=-\nabla V$	(11)

Where $D$ is the electric displacement field, $\rho_{v}$ is the volume charge density, and $V$ is the electric potential.

In the piezoelectric shell, the electric displacement is coupled to the mechanical strain through the piezoelectric constitutive equation:

$D=\varepsilon_{0}E+\varepsilon_{0}\chi E+e: \varepsilon$	(12)

where $\varepsilon_{0}$ is the vacuum electric permittivity and $\chi$ is the relative electrical susceptibility of the material.
